# Development and validation of the AI cognitive offloading scale for Chinese college students

**DOI:** 10.3389/fpsyg.2026.1884676

**Published:** 2026-10-27

**Authors:** Hongying Chang

**Affiliations:** School of Educational Sciences, Minzu Normal University of Xingyi, Xingyi, China

**Keywords:** AI cognitive offloading, assistive offloading, Chinese college students, memory offloading, thinking offloading

## Abstract

**Introduction:**

Against the backdrop of rapid advances in generative artificial intelligence (AI), college students increasingly delegate cognitive tasks to AI systems. This study developed and validated the AI Cognitive Offloading Scale (AI-COS) as an alternative, context-specific operationalization for Chinese college students, focusing on the cognitive functions delegated to AI during learning.

**Methods:**

An initial pool of 26 items was generated through literature review, theoretical analysis, and expert evaluation. Study 1 used data from 665 college students for item analysis and exploratory factor analysis. Study 2 used an independent sample of 1,123 college students for confirmatory factor analysis, reliability testing, criterion-related validity assessment, and measurement invariance testing across gender.

**Results:**

Exploratory factor analysis yielded a 20-item scale with three dimensions: memory offloading, assistive offloading, and thinking offloading. The second-order three-factor model showed satisfactory fit (CFI = 0.951, TLI = 0.944, RMSEA = 0.047, SRMR = 0.036). The scale demonstrated strong internal consistency (Cronbach's α = 0.944), split-half reliability (0.932), and test-retest reliability (0.937). Convergent validity was satisfactory, and full scalar invariance across gender was reported. The total score and all three dimensions were positively associated with AI dependence and negatively associated with metacognitive monitoring. The total score and assistive offloading were negatively associated with need for cognition, whereas memory and thinking offloading were not significantly associated with need for cognition.

**Discussion:**

The findings provide initial evidence for the reliability and validity of the AI-COS. The three dimensions represent distinguishable functions of cognitive delegation rather than a developmental sequence. The scale offers a context-specific instrument for research on AI-mediated cognition, learning regulation, and student development.

## Introduction

Against the backdrop of rapid advances in digital intelligence and generative artificial intelligence (AI), profound transformations are occurring in human knowledge acquisition, learning practices, and the organization of cognitive activity. In particular, generative AI systems represented by large language models (LLMs) have become deeply integrated into information retrieval, text generation, logical reasoning, problem solving, and creative ideation, and are increasingly functioning as important technological tools in college students' daily learning (Dwivedi et al., 2023; Kasneci et al., 2023). Compared with traditional digital tools, generative AI not only provides information but also simulates human language organization, semantic integration, and reasoning processes, thereby exhibiting characteristics of a “cognitive agent.” Consequently, AI-mediated cognitive offloading may occur across multiple functions, from information storage and retrieval to task assistance and higher-order thinking, making it important to distinguish the specific cognitive function being delegated rather than assume that these forms constitute a fixed sequence. Earlier reviews documented applications of artificial intelligence across teaching, learning, and educational administration, providing a broader context for the emergence of generative AI in education (Chen et al., 2020).

Cognitive offloading refers to the use of external tools, environmental cues, or technological media to transfer part of a cognitive task to an external system, thereby reducing internal cognitive load and improving task efficiency (Risko and Gilbert, 2016). From writing systems and calculation devices to internet search engines, human beings have continuously expanded their cognitive capacities through external media. The extended mind theory further proposes that external tools are not merely auxiliary aids to cognition, but may become integral components of the cognitive system itself (Clark and Chalmers, 1998). In traditional contexts, cognitive offloading has primarily involved low-level cognitive activities such as memory storage, information retrieval, and mechanical calculation. However, the emergence of generative AI has fundamentally altered this pattern. Generative AI not only assists individuals in remembering and searching for information, but also directly participates in content generation, logical structuring, viewpoint integration, and solution design—complex cognitive activities traditionally reliant on human cognitive agency (Bubeck et al., 2023). Accordingly, cognitive offloading in generative AI contexts has evolved from outcome-based assistance toward cognitive-process substitution.

This transformation is particularly evident among college students. With the widespread adoption of generative AI tools such as ChatGPT, ERNIE Bot, and Claude, students increasingly rely on AI to support learning tasks in contexts including academic writing, coursework, study planning, coding, and knowledge summarization (Chan and Hu, 2023). Surveys have also examined college students' perceptions and use of generative AI (Baek et al., 2024), including the use of chatbots for assessment assistance and its implications for academic integrity (Gruenhagen et al., 2024). On the one hand, generative AI can improve learning efficiency and reduce the time costs associated with information acquisition and task completion (Zhai et al., 2024). On the other hand, some forms of AI use may reduce active engagement in problem analysis, logical inference, and knowledge integration. Importantly, these possibilities do not imply that all forms of cognitive offloading are equivalent in their psychological meaning or consequences. Memory-related offloading, task-related assistance, and thinking-related offloading may reflect different ways of allocating cognitive responsibility, and their associations with individual characteristics may differ.

From the perspective of cognitive psychology, human beings are inherently inclined to conserve cognitive resources. Limited-capacity theories of cognition suggest that individuals tend to adopt strategies that minimize cognitive effort when confronted with complex tasks, thereby reducing the consumption of psychological resources (Kahneman, 2011). The immediacy, high-quality output, and low usage threshold associated with generative AI further reinforce this tendency toward cognitive economy. Faced with demanding learning tasks, college students may increasingly prefer AI-generated answers, frameworks, or expressions rather than investing greater cognitive resources in independent analysis and processing.

However, research in the learning sciences indicates that genuine knowledge internalization and competence development are not solely founded on efficiency maximization. Bjork and Bjork (2011) theory of desirable difficulties proposes that moderate cognitive effort, retrieval practice, and deep processing are essential conditions for long-term retention and knowledge transfer. In other words, a certain degree of cognitive friction during learning constitutes an important source of capability development. If generative AI excessively replaces cognitive processes that would otherwise require active learner engagement, short-term efficiency gains may come at the cost of diminished development in logical reasoning, problem solving, and metacognitive monitoring.

Existing research has begun to examine the potential influence of AI on cognitive processing. For example, Sparrow et al. (2011) proposed the “Google effect on memory,” suggesting that individuals are less likely to actively remember information when they know it can be externally stored. A subsequent meta-analytical review synthesized research on Internet search behavior and Google effects on memory (Gong and Yang, 2024). Related research showed that saving previously studied information can improve learning and remembering of new information (Storm and Stone, 2015). The source reports a saving-enhanced memory benefit and does not establish that technological dependence impairs deep processing. As generative AI expands from information retrieval to idea generation and cognitive generation, both the depth and scope of cognitive offloading may increase substantially. Consequently, understanding the tension between cognitive economy and deep cognitive processing in generative AI contexts has become a major issue in educational psychology and cognitive psychology.

Although generative AI has emerged as a major topic in educational research, research specifically measuring AI cognitive offloading is still developing. Earlier AI-in-education studies commonly emphasized technology acceptance, usage frequency, perceived usefulness, or technological dependence, whereas more recent work has begun to operationalize cognitive offloading itself. For example, Jiang and Chen (2026) developed and validated an AI-Based Cognitive Offloading (AIBCO) Scale for students in higher education in China, distinguishing lower-order cognitive offloading (e.g., remembering, understanding, and applying) from higher-order cognitive offloading (e.g., analyzing, evaluating, and creating). More recently, Zhu et al. (2026) distinguished dependent cognitive offloading from autonomous cognitive offloading, focusing on whether students delegate core thinking to AI or use AI as a scaffold while retaining cognitive agency. These studies indicate that AI cognitive offloading is no longer an entirely unmeasured phenomenon and that different operationalizations are beginning to emerge. Related research on AI dependence and pedagogical AI overreliance also underscores the importance of distinguishing cognitive delegation from broader patterns of excessive or habitual reliance on AI (Du et al., 2026; Cui et al., 2026).

The present study therefore does not assume that AI cognitive offloading has not previously been measured. Rather, the need is to clarify what aspect of offloading is being operationalized. Existing measures differ in their conceptual focus. The AIBCO Scale of Jiang and Chen (2026) organizes offloading according to the cognitive-demand level represented by Bloom's revised taxonomy, yielding lower-order and higher-order dimensions. Zhu et al. (2026), in contrast, classify offloading according to the manner in which cognitive work is delegated—dependent vs. autonomous—rather than according to the type of cognitive function being offloaded. The AI-COS adopts a different but complementary approach: it classifies AI cognitive offloading according to the primary cognitive function delegated during college learning, distinguishing memory offloading, assistive offloading, and thinking offloading. This distinction is intended to capture differences between using AI as an external repository or retrieval aid, using AI to participate in the execution and organization of academic tasks, and allowing AI to contribute directly to judgment, decision support, and other higher-order thinking activities. Thus, the contribution of the AI-COS lies not in claiming that it is the first measure of AI cognitive offloading, but in providing a three-dimensional, learning-context-specific operationalization that can be used alongside existing content-level and mode-level approaches. This positioning is also consistent with recommendations for rigorous scale development and adaptation, which emphasize explicit construct boundaries, content validity, and empirical cross-validation (Du, 2024).

Accordingly, developing a measurement instrument capable of accurately characterizing the structural features and psychological mechanisms of college students' AI cognitive offloading has become an urgent research priority. Structural measurement of AI cognitive offloading will not only facilitate theoretical clarification of the construct, but also provide an important foundation for understanding transformations in learning practices and cognitive development in the era of generative AI.

Drawing upon distributed cognition theory and transactive memory system (TMS) theory, the present study conceptualizes AI cognitive offloading as a multidimensional form of cognitive delegation rather than as a single technology-use behavior. The framework is informed by prior cognitive-offloading research but is not intended to imply that all forms of AI offloading constitute a single developmental sequence. Instead, the three dimensions are proposed as theoretically distinguishable domains of cognitive function that may be delegated to AI during learning. Distributed cognition theory posits that cognitive activity does not occur exclusively within the individual, but is dynamically distributed across individuals, tools, and environments (Hutchins, 1995; Hollan et al., 2000). Transactive memory system theory further suggests that individuals transfer responsibility for storing certain forms of knowledge to external agents or systems, thereby forming a cognitive division of labor organized around “who knows what” (Wegner, 1987). In research on human work groups, transactive memory systems have been operationalized through specialization, credibility, and coordination (Lewis, 2003). In generative AI contexts, AI systems can function as external cognitive resources across a wider range of activities than traditional memory aids, including information retrieval, task execution, reasoning, and decision support. The present framework therefore focuses on the type of cognitive function being delegated, while recognizing that other approaches may classify AI offloading according to cognitive-demand level or the manner of engagement (Jiang and Chen, 2026; Zhu et al., 2026).

Specifically, the present study conceptualizes three function-based domains of AI cognitive offloading. The first is memory offloading, in which individuals use AI or AI-mediated records for information storage, retrieval, and memorization support. The second is assistive offloading, in which AI participates in the execution, organization, generation, or refinement of learning tasks. The third is thinking offloading, in which AI is used for judgment, decision support, analytical integration, or other higher-order cognitive activities. These dimensions were hypothesized to differ in the type of cognitive responsibility delegated to AI, rather than to represent empirically established stages through which students necessarily progress. This function-based organization complements Jiang and Chen's (2026) lower-/higher-order distinction by providing greater differentiation within the broad domain of academic task support, and it complements Zhu et al. (2026) dependent/autonomous distinction by focusing on what cognitive function is delegated rather than whether the user retains cognitive agency. Accordingly, the AI-COS is positioned as a context-specific operationalization of AI cognitive offloading among college students, with the potential to be used alongside other measures when researchers wish to examine cognitive function, offloading manner, or cognitive-demand level separately.

Within college learning contexts, such transfers of cognitive control may exert profound influences on autonomous learning ability, metacognitive monitoring, and tendencies toward deep cognitive processing. Therefore, understanding AI cognitive offloading from a hierarchical perspective is of considerable importance for revealing emerging characteristics of cognitive development among college students in the era of generative AI.

The study of AI cognitive offloading among college students fundamentally depends upon clarifying its conceptual connotations and constructing a scientifically rigorous measurement instrument. At present, no unified definition of AI cognitive offloading has been established, and its structural dimensions and psychological mechanisms remain insufficiently understood. Accordingly, based on a review of research on cognitive offloading, distributed cognition, and AI-related learning behaviors, the present study seeks to define the construct of AI cognitive offloading among college students and establish its theoretical framework.

The present study defines AI cognitive offloading among college students as a psychological process through which individuals transfer portions of cognitive tasks or cognitive load to generative AI systems during learning in order to complete academic tasks or improve learning efficiency. This process involves not only technology-use behaviors, but also the function-specific redistribution of cognitive responsibilities and the regulation of cognitive agency.

Based on the theoretical framework outlined above, the present study aims to develop and validate the AI Cognitive Offloading Scale (AI-COS) for college students. Specifically, four primary objectives are proposed. First, through literature review, theoretical analysis, and item generation, an initial dimensional structure of AI cognitive offloading among college students will be constructed. Second, through item analysis, exploratory factor analysis, and confirmatory factor analysis, the structural validity of the scale will be examined and its latent dimensions identified. Third, internal consistency reliability, split-half reliability, test-retest reliability, and criterion-related validity will be assessed to evaluate the psychometric properties of the instrument. Fourth, relationships between AI cognitive offloading and metacognitive monitoring, need for cognition, and AI dependence will be examined to assess criterion-related validity. Self-directed learning tendency is not separately measured or reported in the methods/results.

More broadly, the present study seeks not only to develop a psychometric instrument, but also to address a fundamental question concerning the boundaries of human cognition in the era of generative AI. As generative AI increasingly participates in human learning and cognitive processes, understanding which cognitive functions are delegated to AI and how such delegation relates to cognitive agency and learning regulation has become an important issue across educational psychology, learning sciences, and artificial intelligence research. By providing a structured measurement framework for AI cognitive offloading, the present study aims to offer a psychometric instrument and theoretical basis for examining function-specific patterns of AI use in higher education.

## Study 1

### The present study

The present study aimed to develop a preliminary measurement instrument for assessing AI cognitive offloading among college students. Through expert evaluation, item analysis, and exploratory factor analysis (EFA), the initial dimensional structure and psychometric properties of the scale were systematically examined.

### Methods

#### Participants

Participants were recruited from several universities through university student groups and class groups using a convenience sampling approach. The questionnaire was administered online. Following commonly used procedures (Curran, 2016; Xu and Li, 2021), 11 participants with incomplete responses were excluded, resulting in a final valid sample of 665 college students. Among them, 312 were male (46.9%) and 353 were female (53.1%). In terms of academic year, 183 were first-year students (27.5%), 159 were second-year students (23.9%), 172 were third-year students (25.9%), and 151 were fourth-year students (22.7%). The sample size was considered adequate for item analysis and exploratory factor analysis given the initial 26-item pool and the observed sample size. The resulting participant-to-item ratio was approximately 25.6:1, providing a substantial empirical basis for estimating the factor structure.

#### Procedure

For Study 1, the questionnaire was distributed online through student groups and class groups associated with several universities using a convenience sampling approach. Before participation, students were informed of the study purpose, the voluntary nature of participation, confidentiality of responses, and the scientific use of the data. The AI Cognitive Offloading Scale for College Students (AI-COS) was developed through sequential stages. First, the construct definition and provisional dimensional structure were established through literature review and theoretical analysis of cognitive offloading, distributed cognition, transactive memory, and AI-assisted learning. No formal open-ended or semi-structured interviews were conducted, and no separate pilot study was conducted before the main survey. Second, an initial pool of 26 items was generated and refined for clarity, conciseness, conceptual relevance, and appropriateness to college learning contexts. Third, a panel of five experts (all holding Ph.D. degrees in Psychology or Educational Technology, with average professional academic experience exceeding 5 years) independently evaluated all 26 items using a 3-point relevance scale (1 = inappropriate, 2 = appropriate after revision, 3 = appropriate). Their judgments focused on conceptual relevance, representativeness of AI cognitive offloading, wording clarity, and suitability for college learning contexts. The item wording and conceptual fit were revised in response to this evaluation, and the 26-item preliminary questionnaire was retained for empirical evaluation. Fourth, the preliminary questionnaire was evaluated empirically in the Study 1 sample through item discrimination, item-total correlations, and reliability analysis before EFA. Thus, preliminary questionnaire evaluation in the present study refers to expert content evaluation followed by empirical item analysis rather than to a separate pilot sample. A 5-point Likert response format ranging from “strongly disagree” to “strongly agree” was used. Finally, SPSS 26.0 and Mplus 8.3 were used for item analysis and EFA.

### Results

#### Preliminary questionnaire evaluation and item analysis

The 26-item preliminary questionnaire was first evaluated empirically using item discrimination, item-total correlations, and the effect of item deletion on internal consistency. For item discrimination, participants were divided into high-score and low-score groups based on the top and bottom 27% of total scores, followed by independent-samples *t* tests. The absolute t values for the 26 items ranged from 14.32 to 27.05 (*p* < 0.001), indicating satisfactory discrimination across all items. Item homogeneity was further examined using item-total correlations, which ranged from 0.57 to 0.75 and all exceeded the prespecified criterion of 0.40. In addition, deleting any individual item did not substantially increase the overall Cronbach's α coefficient (α = 0.948). These findings indicated that all 26 items met the preliminary empirical screening criteria and were retained for EFA. Because no separate pilot sample was used, these analyses constitute the empirical evaluation of the preliminary questionnaire in the present study. The item-analysis results for all 26 initial items are presented in Table 1.

**Table 1:** Results of item analysis for the AI cognitive offloading scale (initial 26 items).

Item	Mean (M)	Standard deviation (SD)	Item discrimination (*t*)^***^	Correlation between each item and total (*r*)	Delete Cronbach's α after this item
1	2.73	0.973	15.560	0.572	0.948
2	2.84	1.013	16.762	0.607	0.948
3	2.80	0.976	22.278	0.665	0.947
4	2.70	0.965	17.059	0.618	0.948
5	2.78	0.969	18.626	0.622	0.948
6	2.96	0.965	27.051	0.733	0.946
7	2.97	0.968	25.790	0.725	0.946
8	2.88	0.981	26.545	0.723	0.946
9	2.86	0.966	23.875	0.714	0.947
10	3.11	0.969	23.328	0.704	0.947
11	3.02	1.001	26.540	0.742	0.946
12	3.13	0.972	21.172	0.686	0.947
13	2.79	0.943	26.667	0.751	0.946
14	2.83	0.985	24.158	0.698	0.947
15	3.07	0.997	24.001	0.712	0.947
16	2.94	0.965	22.271	0.684	0.947
17	2.70	0.919	24.436	0.720	0.947
18	2.87	0.969	20.177	0.623	0.948
19	2.72	0.946	18.216	0.638	0.947
20	2.84	0.968	18.879	0.647	0.947
21	2.84	0.932	20.943	0.665	0.947
22	3.22	0.974	15.441	0.585	0.948
23	2.99	0.993	14.324	0.577	0.948
24	2.86	0.955	23.191	0.686	0.947
25	2.78	0.969	15.571	0.572	0.948
26	3.21	0.938	14.580	0.584	0.948

*N* = 665. ^***^p < 0.001. The overall scale Cronbach's α = 0.948.

#### Exploratory factor analysis

Principal axis factoring (PAF) with direct oblimin rotation was used to examine the latent factor structure of the 26-item preliminary questionnaire in Sample 1 (*N* = 665). PAF was selected because the purpose of the analysis was to identify latent common factors underlying the observed item responses rather than to reduce the observed variables to a smaller set of components. Direct oblimin rotation was selected because memory offloading, assistive offloading, and thinking offloading were theoretically expected to be related rather than statistically independent. The number of factors was evaluated using multiple sources of evidence, including eigenvalues greater than 1, scree-plot inspection, cumulative variance explained, and conceptual interpretability. Item retention was subsequently considered using factor-loading magnitude, cross-loading patterns, and conceptual relevance.

For the initial 26-item pool, the KMO value was 0.965 and Bartlett's test of sphericity was significant, χ^2^(325) = 8904.89, *p* < 0.001, indicating strong inter-item correlations and excellent sampling adequacy. PAF with direct oblimin rotation was then used to extract latent factors. Based on the eigenvalue-greater-than-one criterion, scree-plot inspection, cumulative variance explained (54.68%), and interpretability of the factor solution, a three-factor solution was retained. The original analysis did not include a formal univariate normality test based on skewness and kurtosis, and no such statistics were used as item-retention criteria. Accordingly, the revised manuscript does not introduce unreported normality coefficients. The factor-analytic decision was instead based on the sampling-adequacy tests, extraction and rotation procedures described above, factor-retention evidence, and item-level screening criteria.

During item purification, both statistical criteria and content validity considerations were taken into account. First, three original items with factor loadings below the prespecified retention threshold of 0.40 (W16, W23, and W25) were removed. This threshold was used as an item-retention criterion and should be distinguished from the reporting convention in Table 2. Subsequently, to improve parsimony and conceptual clarity, three semantically redundant items under Factor 1 (W8, W9, and W10) were further eliminated following semantic evaluation. The final version retained 20 items. The pattern matrix results (see Table 2) indicated that all retained items exhibited satisfactory factor loadings ranging from 0.447 to 0.750, with no substantial cross-loadings observed. Based on the semantic characteristics of the retained items, the three factors were labeled memory offloading, assistive offloading, and thinking offloading. These dimensions represent distinguishable domains of AI cognitive offloading in digital learning contexts. Their ordering is descriptive of the cognitive functions represented by the retained items and should not be interpreted as evidence of a developmental or progressive sequence.

**Table 2:** Results of exploratory factor analysis and factor loadings for the cognitive offloading scale (*N* = 665).

Item	Memory offloading	Assistive offloading	Thinking offloading
Item 1	0.628		
Item 2	0.596		
Item 3	0.584		
Item 4	0.555		
Item 5	0.539		
Item 6		0.677	
Item 7		0.673	
Item 8		0.668	
Item 9		0.604	
Item 10		0.597	
Item 11		0.579	
Item 12		0.528	
Item 13		0.519	
Item 14		0.506	
Item 15		0.447	
Item 16			0.750
Item 17			0.710
Item 18			0.629
Item 19			0.546
Item 20			0.456
Eigenvalue	11.542	1.506	1.168
Variance contribution rate (%)	44.394%	5.791%	4.493%
Cumulative variance contribution rate (%)	44.394%	50.184%	54.677%

Item numbers refer to the final 20-item scale.

Factor loadings below 0.40 are not displayed in the pattern matrix for clarity; this is a reporting convention and does not mean that values below 0.40 were merely hidden during item screening. Items removed during item purification are not included in this table. The 0.40 loading criterion was applied to item retention, and three items (W16, W23, and W25) were removed on this basis. Three additional items (W8, W9, and W10) were removed because of semantic redundancy. Extraction method: Principal Axis Factoring. Direct oblimin rotation was used because the factors were expected to be correlated. Because the rotation direction can change the sign of factor loadings, absolute loading values are reported.

According to the screening rules of factor analysis, the item was analyzed and screened. The research followed the following principles when selecting the topics (Comrey and Lee, 2013; Wang, 2014).

- The factor loading of each retained item on its corresponding factor was required to exceed 0.40. The 0.40 threshold was applied to the item-retention decision, whereas Table 2 displays only loadings of 0.40 or above for readability.
- Items were expected to show no substantial cross-loadings across factors.
- Each retained factor was required to contain at least three items to support a stable and interpretable factor definition.
- The substantive content and conceptual attribution of each item were examined to ensure that the retained items adequately represented the intended construct.

The EFA results supported a three-dimensional structure of AI cognitive offloading. Memory offloading, assistive offloading, and thinking offloading emerged as relatively distinct yet interrelated dimensions. The factor solution therefore supports functional differentiation within the construct, while not establishing that the dimensions constitute stages or a temporal sequence.

The three-factor structure should therefore be understood as a multidimensional representation of different functions that may be delegated to AI rather than as a hierarchical or developmental pathway. Because the EFA is cross-sectional, the factor solution cannot determine whether students progress from memory offloading to assistive or thinking offloading over time.

Through the EFA-based item purification procedure, the formal version of the scale was ultimately established as a 20-item instrument comprising three dimensions.

The first factor, memory offloading, consisted of five items (Item 1–Item 5). These items reflected students' tendencies to delegate the storage, retrieval, and memorization of basic learning information to AI systems, thereby transferring part of the cognitive burden to external technological tools. Exploratory factor analysis yielded loadings of 0.539–0.628 for memory offloading (Table 2).

The second factor, assistive offloading, consisted of 10 items in the final scale (Items 6–15). These items captured students' reliance on AI for logical analysis, content generation, structural organization, and the completion of complex academic tasks, reflecting the delegation of task-related cognitive processing to AI. Within this dimension, the retained items represent a coherent set of assistive functions.

The third factor, thinking offloading, consisted of five items in the final scale (Items 16–20). These items primarily reflected the tendency to rely on AI for judgment, decision support, and cognitive guidance. The label refers to the cognitive function represented by these items and does not imply that this form of offloading is necessarily more advanced, less adaptive, or developmentally later than the other dimensions.

During the item purification stage, six of the original 26 items were removed due to insufficient factor loadings or semantic redundancy, including the original Items 8, 9, 10, 16, 23, and 25. After removal, the retained items were renumbered consecutively for the final 20-item scale: memory offloading (Items 1–5), assistive offloading (Items 6–15), and thinking offloading (Items 16–20). The final AI-COS therefore uses a single, consistent numbering system across the complete item table, factor descriptions, and subsequent analyses.

The content of the three factors can be summarized by representative cognitive functions and item keywords. Memory offloading includes storing, retrieving, and memorizing learning information (e.g., “store,” “retrieve,” and “memorize”). Assistive offloading includes AI-supported analysis, content generation, summarization, and task completion (e.g., “analyze,” “generate,” “summarize,” and “complete”). Thinking offloading includes reliance on AI for judgment, decision support, planning, and cognitive guidance (e.g., “judgment,” “decision,” “study plan,” and “guidance”). These examples are intended to illustrate the conceptual coverage of each factor rather than reproduce the complete questionnaire, which is provided in Table 3.

**Table 3:** Complete item content of the AI cognitive offloading scale.

Dimension	Item	Item wording
Memory offloading	1	I habitually delegate information that needs to be remembered during learning directly to AI, rather than attempting to memorize it in my mind.
	2	I have become increasingly reliant on AI to store important materials for me, as I find it more reliable than my own memory.
	3	When encountering previously learned knowledge, I tend to search AI history records rather than first attempting to recall it myself.
	4	I feel there is no need to expend effort memorizing information that AI can retrieve for me anytime and anywhere.
	5	Rather than exercising my own memory, I prefer to use electronic devices or AI to share the memory burden.
Assistive offloading	6	When facing difficult problems, I am more accustomed to having AI provide complete answers directly rather than breaking down the steps myself.
	7	I habitually let AI analyze complex logic for me, rather than clarifying my own thoughts independently.
	8	I frequently delegate tedious learning tasks to AI for processing.
	9	When completing assignments, papers, and the like, I habitually use AI to generate content or drafts first, then make only minor revisions.
	10	I tend to use AI to obtain results quickly, rather than mastering the process through self-directed learning.
	11	I habitually have AI summarize the key points of books or materials for me, and I rarely read through the original texts myself.
	12	When confronted with difficult-to-digest learning materials, I rely heavily on AI to convert them into easily understandable simplified versions.
	13	In the current learning environment, I believe that moderate reliance on AI is an inevitable trend.
	14	Compared with traditional independent study, I think that collaborative learning with AI represents true efficiency.
	15	I believe that delegating a portion of complex academic tasks to AI is not a form of laziness, but rather a smart strategy.
Thinking offloading	16	Rather than trusting my own intuition or judgment, I am more willing to trust the analytical results provided by AI.
	17	When hesitating among multiple options, I tend to let AI make the final decision for me.
	18	I habitually have AI create study plans for me and strictly follow the steps it provides.
	19	When I am unsure about what to do, I habitually adopt AI's suggestions as the guiding principle for my actions.
	20	I believe that the learning suggestions and answers provided by AI are generally scientific and trustworthy.

## Study 2

### The present study

To verify the reliability and validity of the structure and scale obtained by the exploratory factor analysis, Study 2 recruited a new and independent sample of college students to conduct confirmatory factor analysis, reliability analysis, validity analysis, and measurement-invariance analysis. The independent-sample design was intentional: Study 1 was used for item purification and exploratory identification of the factor structure, whereas Study 2 was reserved for confirmatory testing and subsequent psychometric evaluation. This approach reduces the risk of capitalizing on chance in the exploratory sample and provides a cross-sample test of the proposed structure.

### Methods

#### Participants

Participants for Study 2 were recruited from several universities through university student groups and class groups using the same convenience sampling approach as in Study 1. The questionnaire was administered online. Referring to the commonly used method (Curran, 2016; Xu and Li, 2021), 58 participants whose answers were incomplete were excluded, leaving 1,123 valid participants. The final sample consisted of 1,123 valid participants, including 563 males (50.1%) and 560 females (49.9%). Regarding academic standing, 288 participants were first-year students (25.6%), 290 were second-year students (25.8%), 269 were third-year students (24.0%), and 276 were fourth-year students (24.6%).

#### Tools

##### AI cognitive offloading scale for college students

The AI-COS was the primary instrument developed and validated in the present study. It contains 20 items across three dimensions: memory offloading, assistive offloading, and thinking offloading, rated on a 5-point Likert scale from “completely disagree” to “completely agree.” Higher scores indicate stronger AI cognitive offloading. In Study 2, the scale was used to evaluate its factor structure, reliability, criterion-related validity, and measurement invariance across gender.

##### Student generative AI dependence questionnaire

The questionnaire developed by Yu and Yan (2026) was included as an external criterion measure. It contains 17 items across cognitive-behavioral dependence, functional dependence, and emotional dependence. It was used to examine whether AI cognitive offloading was associated with broader dependence on generative AI. In the present study, Cronbach's α was 0.916.

##### Need for cognition scale

Need for cognition concerns the tendency to engage in and enjoy effortful thinking (Cacioppo and Petty, 1982), and brief instruments include a six-item version developed by Coelho et al. (2020). A four-item measure was used to assess need for cognition (Wu et al., 2023) as an individual-difference criterion measure. All items were rated on a 5-point Likert scale from “completely disagree” to “completely agree,” with higher scores indicating a stronger need for cognition. It was used to examine whether students' tendency to engage in and enjoy cognitively demanding activity was associated with AI cognitive offloading. In the present study, Cronbach's α was 0.812.

##### Metacognitive monitoring scale

The scale originally developed by Kang (2005) was included as a criterion measure of students' metacognitive monitoring. It contains 6 items rated on a 5-point scale from 1 (“never”) to 5 (“always”), with higher scores indicating stronger metacognitive monitoring. It was used to examine whether AI cognitive offloading was associated with students' monitoring and regulation of their own learning processes. In the present study, Cronbach's α was 0.810.

#### Procedure

For Study 2, the questionnaire was distributed online through student groups and class groups associated with several universities, using the same convenience-sampling procedure as in Study 1. The Study 2 participants were newly recruited and did not overlap with the Study 1 sample. Before completing the questionnaire, participants were informed that participation was voluntary, responses would remain confidential, and the data would be used only for scientific research. After data collection, Mplus 8.3 was used to conduct confirmatory factor analysis, whereas SPSS 26.0 was used to examine reliability and validity indices.

### Results

#### Confirmatory factor analysis

To examine the theoretical structure of the scale, three competing models were compared (see Table 4): (a) a one-factor model (M1); (b) a first-order three-factor model (M2), including memory offloading, assistive offloading, and thinking offloading; and (c) a second-order factor model (M3), in which AI cognitive offloading was specified as a higher-order latent construct underlying the three first-order dimensions. The second-order specification was theoretically motivated by the definition of AI cognitive offloading as an overarching process in which students delegate different types of cognitive functions to generative AI. Thus, the three dimensions were conceptualized as functionally distinct but correlated manifestations of a common higher-order construct, rather than as independent constructs or developmental stages. This specification also permits the use of a total AI cognitive offloading score while preserving the distinct interpretation of the three dimensions. As reported in Table 4, the one-factor model yielded CFI = 0.886, TLI = 0.873, RMSEA = 0.071, and SRMR = 0.051. The first-order three-factor model yielded CFI = 0.940, TLI = 0.932, RMSEA = 0.052, and SRMR = 0.158. The second-order model yielded CFI = 0.951, TLI = 0.944, RMSEA = 0.047, and SRMR = 0.036 and was used for the subsequent analyses. The substantially stronger fit of M3, together with the significant second-order loadings reported in Table 5, provides empirical support for representing the three dimensions under a common higher-order AI cognitive offloading construct.

**Table 4:** Evaluation of fit indices for each competing model (*N* = 1,123).

Model	χ^2^	*df*	CFI	TLI	RMSEA	SRMR	*p*
M1	1140.766	170	0.886	0.873	0.071	0.051	< 0.001
M2	628.228	169	0.940	0.932	0.052	0.158	< 0.001
M3	589.265	167	0.951	0.944	0.047	0.036	< 0.001

χ^2^, chi-square statistic; *p* values are based on the chi-square distribution with the reported degrees of freedom. All model chi-square tests were significant at *p* < 0.001.

**Table 5:** Summary of loadings, reliability, and AVE results for each dimension of the scale (*N* = 1123).

Latent factor	Item	λ	*t*	CR	AVE	Second-order load (γ)
Memory offloading	Items 1–5	0.638–0.749	24.58–41.10	0.840	0.501	0.868
Assistive offloading	Items 6–15	0.598–0.783	23.85–53.08	0.906	0.507	0.964
Thinking offloading	Items 16–20	0.733–0.786	38.50–48.04	0.869	0.570	0.866

Item numbers refer to the final 20-item scale.

#### Composite reliability and convergent validity

Based on the second-order factor model (M3), the psychometric properties of the scale were further examined (see Table 5).

First, all items demonstrated satisfactory standardized factor loadings (λ), ranging from 0.598 to 0.786. In addition, all factor loadings were highly significant, with *t* values ranging from 23.85 to 53.08 (*p* < 0.001), indicating strong associations between observed indicators and their corresponding latent dimensions. Composite reliability (CR) analyses demonstrated satisfactory internal consistency across dimensions. Specifically, the CR values for memory offloading, assistive offloading, and thinking offloading were 0.840, 0.906, and 0.869, respectively. Regarding convergent validity, the AVE values for the three dimensions were 0.501, 0.507, and 0.570, respectively. Furthermore, the higher-order factor analysis showed that the loadings of the three first-order dimensions on the second-order AI cognitive offloading construct were 0.868 for memory offloading, 0.964 for assistive offloading, and 0.866 for thinking offloading, all *p* < 0.001. These findings indicate that the three dimensions are strongly related to the overarching construct and support the interpretation of AI cognitive offloading as a higher-order multidimensional construct. Accordingly, use of the total scale score is supported, while the three dimension scores remain interpretable as function-specific components.

#### Discriminant validity

Discriminant validity was considered in relation to the conceptual and empirical distinctness of the three first-order dimensions. The three-factor CFA model distinguished memory offloading, assistive offloading, and thinking offloading, while the second-order model indicated that these dimensions were strongly related manifestations of a broader AI cognitive offloading construct. The AVE values were 0.501, 0.507, and 0.570 for memory, assistive, and thinking offloading, respectively. However, the original analysis did not retain a latent-factor correlation matrix or calculate a heterotrait-monotrait ratio (HTMT), and these statistics cannot be reconstructed reliably from the currently reported model-fit indices, standardized loadings, CR, and AVE values alone. Therefore, the present study does not claim that strict statistical discriminant validity between the three highly related dimensions has been fully established. Instead, the evidence supports their empirical differentiation as distinct but related functions within a higher-order construct. Future validation studies should directly report latent-factor correlations and HTMT or another established discriminant-validity criterion using item-level data.

#### Reliability analysis

Internal consistency was assessed using Cronbach's alpha (α), which estimates the extent to which items within each scale or subscale show consistent responses. Split-half reliability was assessed using the split-half procedure in SPSS 26.0 to provide a complementary estimate of internal consistency by examining the consistency between two halves of the item set. Test-retest reliability was assessed using the association between scores obtained at the initial assessment and at a 1-month follow-up, providing evidence of temporal stability. These procedures were selected to evaluate complementary aspects of reliability: internal coherence of the items, consistency across item halves, and stability over time. As shown in Table 6, the overall scale demonstrated excellent internal consistency (α = 0.944) and split-half reliability (0.932). The Cronbach's α coefficients of the three subscales ranged from 0.834 to 0.912, whereas split-half reliability coefficients ranged from 0.845 to 0.910. A total of 99 college students completed the scale again after a 1-month interval. The test-retest reliability coefficient for the total scale was 0.937, while the coefficients for the three dimensions ranged from 0.833 to 0.899, indicating good temporal stability. In the Composite reliability and convergent validity section, specify that the loadings are CFA estimates: The standardized CFA loadings ranged from 0.638 to 0.749 for memory offloading, 0.598 to 0.783 for assistive offloading, and 0.733 to 0.786 for thinking offloading (Table 5).

**Table 6:** Reliability indices of the AI cognitive offloading scale.

Reliability indices	Memory offloading	Assistive offloading	Thinking offloading	AI cognitive offloading
Cronbach α coefficient	0.834	0.912	0.871	0.944
Split-half reliability	0.845	0.910	0.855	0.932
Test-retest reliability	0.859	0.899	0.833	0.937

Internal consistency and split-half reliability were assessed in the Study 2 sample (*N* = 1,123); test-retest reliability was assessed in the retest sample (*n* = 99).

#### Content validity

Content validity was evaluated during item development through independent expert review. Five university professors with expertise in psychology and educational technology rated each of the initial 26 items on a 3-point relevance scale: 1 = inappropriate, 2 = appropriate after revision, and 3 = appropriate. Expert judgments focused on conceptual relevance, representativeness of AI cognitive offloading, clarity of wording, and suitability for college learning contexts. Items were revised in response to expert feedback, and all 26 items were retained for the subsequent empirical item-analysis stage. Because the original expert-review records were used primarily for item-level screening and wording refinement and no formal item-level content validity index (I-CVI) or scale-level content validity index (S-CVI) was calculated at that stage, the present study does not report numerical CVI values. The subsequent factor-analytic evidence was therefore used together with the expert review to evaluate content and structural adequacy.

#### Criterion-related validity

The widespread adoption of artificial intelligence has become an important factor influencing college students' learning and cognitive development. Previous research suggests that AI-assisted cognitive processes are closely associated with technological dependence, cognitive motivation, and metacognitive regulation. Accordingly, the AI Dependence Scale, Metacognitive Monitoring Scale, and Need for Cognition Scale were used as criterion measures. As shown in Table 7, the total score of the AI Cognitive Offloading Scale was significantly and positively correlated with AI dependence (*r* = 0.619, *p* < 0.001), while the memory, assistive, and thinking offloading dimensions were also significantly and positively correlated with AI dependence (*r* = 0.489, 0.593, and 0.567, respectively; all *p* < 0.001). The total score and all three dimensions were significantly and negatively correlated with metacognitive monitoring (*r* = −0.234, −0.245, −0.211, and −0.180, respectively; all *p* < 0.001). In relation to need for cognition, the total score (*r* = −0.076, *p* = 0.011) and assistive offloading (*r* = −0.121, *p* < 0.001) showed significant negative correlations, whereas memory offloading (*r* = −0.046, *p* = 0.120) and thinking offloading (*r* = 0.006, *p* = 0.841) were not significantly correlated with need for cognition. These differentiated association patterns provide initial criterion-related validity evidence for the AI Cognitive Offloading Scale.

**Table 7:** Criterion-related validity correlations for the AI cognitive offloading scale.

Variable	AI cognitive offloading (total)	Memory offloading	Assistive offloading	Thinking offloading
AI dependence	0.619^***^	0.489^***^	0.593^***^	0.567^***^
Metacognitive monitoring	−0.234^***^	−0.245^***^	−0.211^***^	−0.180^***^
Need for cognition	−0.076^*^	−0.046	−0.121^***^	0.006

Pearson correlations; *N* = 1,123. ^*^p < 0.05; ^***^p < 0.001. Correlations were tested using two-tailed Pearson correlation analyses.

#### Measurement invariance across gender

Multigroup confirmatory factor analysis (MG-CFA) was conducted to examine measurement invariance across gender.

Following the recommendations of Chen (2007), configural invariance (M1), metric invariance (M2), and scalar invariance (M3) models were sequentially tested. Changes in fit indices (ΔCFI ≤ 0.010, ΔTLI ≤ 0.010, and ΔRMSEA ≤ 0.015) were used as evaluation criteria. As shown in Table 8, the configural invariance model (M1) demonstrated satisfactory fit, χ^2^ = 12685.670, df = 380, CFI = 0.940, TLI = 0.932, RMSEA = 0.063, and SRMR = 0.040, indicating that the three-factor structure was applicable across male and female groups. Next, in the metric invariance model (M2), factor loadings were constrained to equality across groups. The model fit showed no substantial deterioration (ΔCFI = 0.001, ΔTLI = 0.004, ΔRMSEA = −0.008, ΔSRMR = 0.002), supporting metric invariance. Finally, in the scalar invariance model (M3), item intercepts were additionally constrained to equality across groups. The model fit remained stable (ΔCFI = −0.001, ΔTLI = 0.002, ΔRMSEA = 0.005, ΔSRMR = 0.001), thereby supporting scalar invariance. Overall, the findings demonstrated full scalar invariance across gender, indicating that the scale possesses equivalent psychometric properties for male and female participants and providing a reliable basis for subsequent latent mean comparisons across gender groups.

**Table 8:** Fit indices and testing for measurement invariance across groups.

Model	χ^2^	*df*	CFI	TLI	RMSEA	SRMR	ΔCFI	ΔTLI	ΔRMSEA	ΔSRMR	*p*
M1: configural	1067.006	334	0.940	0.932	0.063	0.040	–	–	–	–	< 0.001
M2: metric	1079.183	351	0.941	0.936	0.055	0.042	+0.001	+0.004	−0.008	+0.002	< 0.001
M3: scalar	1105.254	368	0.940	0.938	0.060	0.043	−0.001	+0.002	+0.005	+0.001	< 0.001

χ^2^, chi-square statistic; *p* values are based on the chi-square distribution with the reported degrees of freedom. All model chi-square tests were significant at *p* < 0.001. Changes in fit indices were calculated from the displayed values by subtracting the preceding model's value from the current model's value.

## Discussion

The present study developed and validated the AI Cognitive Offloading Scale for College Students (AI-COS) by examining how different cognitive functions are delegated to generative AI in learning contexts. The contribution should be understood as an alternative and context-specific operationalization rather than as the first measurement of AI cognitive offloading. Recent work has already provided complementary approaches. Jiang and Chen (2026) developed the AIBCO Scale for higher-education students in China, distinguishing lower-order from higher-order cognitive offloading according to the level of cognitive demand. Zhu et al. (2026) distinguished dependent from autonomous cognitive offloading according to whether users substitute their own thinking or use AI as a scaffold while retaining cognitive agency. In contrast, the AI-COS organizes offloading by the primary cognitive function delegated to AI—memory, assistive task-related processing, and thinking—and was developed specifically for Chinese college students' learning activities. These distinctions make the AI-COS conceptually complementary to existing measures: the AIBCO framework emphasizes cognitive-demand level, the dependent/autonomous framework emphasizes the manner of delegation, and the AI-COS emphasizes the function being delegated. This function-based perspective may be useful when researchers need to distinguish information storage and retrieval from AI participation in academic task execution and from delegation of judgment or decision support.

From a theoretical standpoint, the findings suggest that the influence of generative AI on college students' learning is no longer confined to information provision or efficiency enhancement. Rather, AI systems can be implicated in task execution, cognitive processing, and intellectual regulation, functioning as components of an extended cognitive system. The extended mind theory argues that external tools are not merely ancillary to cognition, but may become constitutive elements of the cognitive system itself (Clark and Chalmers, 1998). Likewise, cognitive offloading research indicates that external resources can reduce internal cognitive load, while the consequences of offloading may depend on the nature and regulation of the delegated activity (Risko and Gilbert, 2016; Turner, 2022). Within this framework, the present study conceptualized three functionally distinct forms of AI cognitive offloading—memory offloading, assistive offloading, and thinking offloading. These dimensions describe different cognitive functions that may be delegated to AI; the present cross-sectional factor structure does not establish that students move from one form to another over time.

One of the important findings is that AI cognitive offloading among college students can be differentiated into three interrelated dimensions: memory offloading, assistive offloading, and thinking offloading. Importantly, this three-dimensional structure represents one empirically supported operationalization of the construct rather than a claim that these are the only possible dimensions. The exploratory factor analysis yielded a clear factor pattern, and the confirmatory factor analysis supported a higher-order structure. The resulting dimensions are also conceptually distinguishable from recent alternatives. Jiang and Chen (2026) separated AI-based cognitive offloading into lower-order and higher-order forms, while Zhu et al. (2026) distinguished dependent and autonomous offloading according to the degree to which users retain cognitive agency. The AI-COS instead differentiates the functions delegated to AI within college learning: memory-related storage and retrieval, task-related assistance and execution, and higher-order thinking-related judgment and decision support. The three approaches therefore address related but non-identical questions about AI-mediated cognition.

Within this framework, memory offloading primarily reflects students' tendency to delegate basic cognitive operations—such as memorization, retrieval, storage, and organization—to AI systems, thereby transferring part of the cognitive burden to external technological resources. This dimension is closely aligned with the classic notion of cognitive offloading, namely the use of external media to reduce internal cognitive costs. Assistive offloading, in contrast, involves AI-supported completion and organization of learning tasks, including content generation, textual organization, expression refinement, and the handling of complex academic assignments. Thinking offloading involves the delegation of judgment support, analytic integration, logical inference, and decision assistance. These dimensions therefore differ in the type of cognitive function being delegated, but the present factor analysis does not demonstrate that they form a developmental continuum, that one dimension necessarily precedes another, or that greater endorsement of one dimension causes movement toward another. Their co-occurrence under a higher-order factor indicates shared variance in AI cognitive offloading, not temporal progression.

The confirmatory factor analysis further supported the proposed multidimensional structure. The second-order model provided the best fit among the three competing specifications, and the three first-order dimensions showed strong loadings on the higher-order AI cognitive offloading factor. This pattern supports a hierarchical representation in which memory offloading, assistive offloading, and thinking offloading are distinguishable functions that share substantial common variance. The model therefore provides a statistical basis for reporting both dimension-specific scores and an overall AI cognitive offloading score. Importantly, the higher-order representation should not be interpreted as evidence that the three dimensions form a temporal sequence or a developmental hierarchy.

The criterion-related validity findings are particularly informative because they reveal differentiated association patterns across dimensions. The total AI cognitive offloading score and all three dimensions showed significant positive associations with AI dependence, indicating that greater reported AI cognitive offloading was associated with greater AI dependence across the three function-based forms. Similarly, the total score and all three dimensions were negatively associated with metacognitive monitoring. In relation to need for cognition, the total score and assistive offloading were negatively associated with need for cognition, whereas the memory and thinking offloading dimensions were not significantly related to this criterion. These findings should not be interpreted as evidence that cognitive offloading is inherently beneficial or detrimental. Instead, they suggest that the psychological meaning of offloading may depend on which cognitive function is delegated, the extent of delegation, and the degree to which learners continue to monitor and regulate the process.

From a functional perspective, cognitive offloading may enhance cognition when external resources reduce unnecessary cognitive demands, support information organization and retrieval, or free internal resources for monitoring, integration, and other higher-order activities (Risko and Gilbert, 2016; Dunn and Risko, 2016). In generative AI contexts, such benefits may include faster access to information, assistance with organizing complex materials, and support for task execution. However, offloading may also diminish aspects of cognitive functioning when external systems replace processes that learners need to actively practice. Excessive delegation of retrieval, analysis, judgment, or decision making may reduce opportunities for retrieval practice, deep processing, independent problem solving, and the development of cognitive agency. This possibility is consistent with the learning principle that some degree of cognitive effort and retrieval practice contributes to durable learning (Bjork and Bjork, 2011). Importantly, the present findings do not establish that assistive or thinking offloading causes cognitive impairment, nor do they demonstrate that memory offloading necessarily produces cognitive benefits. Rather, the contrasting correlational patterns indicate that different forms of offloading may have different functional implications. A balanced interpretation therefore treats AI cognitive offloading as a resource-allocation strategy whose effects may range from cognitive support to cognitive substitution depending on the task, the function delegated, and the learner's metacognitive regulation. Future longitudinal and experimental studies should directly test when offloading preserves or enhances cognitive performance and when it may undermine learning, cognitive independence, or metacognitive control. Research has also reported associations among AI tool use, cognitive offloading, and critical thinking (Gerlich, 2025), providing a further rationale for examining these relationships longitudinally.

This interpretation is also consistent with the broader literature on cognitive offloading, in which externalization can be useful when it is strategically selected to manage cognitive demands, while the consequences may differ when external tools replace processes that learners need to actively practice. Appropriate memory offloading—for example, using AI to organize information or support factual retrieval—may, under some conditions, allow cognitive resources to be allocated to monitoring, integration, or other learning activities. At the same time, assistive and thinking offloading may also be used strategically, and their positive associations with AI dependence in the present study should not be interpreted as proof that these forms are inherently maladaptive. The key implication is that the psychological meaning of an offloading behavior cannot be inferred from its position on an assumed progression; it depends on what function is delegated, how the learner monitors the process, and whether cognitive agency is retained. Future longitudinal and experimental studies should directly test whether different offloading functions predict changes in learning, metacognitive regulation, or AI dependence over time. Experimental research on text learning has shown that more accurate metacognitive monitoring can improve the regulation of study and subsequent learning (Thiede et al., 2003).

A meta-analysis has linked need for cognition to academic achievement, supporting its relevance as an individual-difference variable in educational research (Liu and Nesbit, 2024). The negative association between need for cognition and total AI cognitive offloading, together with the negative association observed for assistive offloading, further indicates that individual differences in willingness to engage in cognitive effort may be relevant to some forms of AI-related delegation. However, the absence of significant associations between need for cognition and memory or thinking offloading suggests that this relationship is not uniform across all dimensions. Students who enjoy complex thinking and are more willing to invest cognitive effort may report lower overall tendencies toward AI cognitive offloading, but the present cross-sectional data do not establish that cognitive effort prevents offloading or that offloading reduces tolerance for cognitive complexity. Overall, the findings support a differentiated view of AI cognitive offloading in which cognitive function, metacognitive monitoring, AI dependence, and need for cognition are related but non-equivalent aspects of students' interaction with AI.

The present study also examined measurement invariance across gender, and the results supported configural, metric, and scalar invariance. This indicates that the scale measures the same underlying construct among male and female students rather than different psychological attributes, thereby providing a valid methodological basis for future gender comparisons. This is especially important for a new psychometric instrument, because a high-quality scale must not only demonstrate reliability and validity, but also function equivalently across groups. More broadly, these findings align with an increasingly emphasized principle in higher education AI governance: policy and intervention should focus not merely on whether students use AI, but on how they use AI, for which tasks they rely on AI, and whether different student groups show distinct patterns of cognitive offloading. Analyses of AI governance policies in U.S. higher education similarly suggest a shift from simple restriction toward guided integration, with growing attention to assignment-level and course-level recommendations for appropriate AI use (McDonald et al., 2025).

From a practical and theoretical perspective, the present study has implications for AI governance and educational practice in higher education. The findings suggest that guidance should focus not merely on whether students use AI, but on which cognitive functions are delegated and how students regulate that delegation. Memory offloading, assistive offloading, and thinking offloading may require different forms of instructional attention because they involve different cognitive functions. In particular, thinking offloading can involve delegating judgment, reasoning, integration, analysis, and decision-making to AI systems. Recent research on dependent and autonomous cognitive offloading suggests that when users delegate core thinking rather than use AI as a scaffold while retaining responsibility for evaluating information and making decisions, cognitive agency can become more directly implicated (Zhu et al., 2026). This provides a theoretical basis for considering thinking offloading as especially relevant to cognitive agency, while not implying that it is a later developmental stage or inherently harmful. At the same time, the present cross-sectional data do not establish that thinking offloading causes a reduction in cognitive agency. Educational interventions may therefore benefit from emphasizing metacognitive monitoring, transparency about AI involvement, and preservation of appropriate cognitive agency across different task types. AI literacy education may complement these efforts by helping students critically evaluate and appropriately use AI systems (Long and Magerko, 2020).

More broadly, the present study contributes to theoretical discussions concerning the boundary between human cognition and artificial intelligence by providing a context-specific framework for describing the cognitive functions delegated to AI. Generative AI systems can participate in information retrieval, task execution, reasoning, and decision support, but the way these functions are conceptualized and measured may vary across studies. The AI-COS should therefore be interpreted as complementary to, rather than replacing, existing AI cognitive offloading measures. Its particular contribution is to provide a three-dimensional, college-learning-oriented operationalization that distinguishes memory-related offloading, assistive task-related offloading, and thinking-related offloading. This function-based differentiation also provides a more precise basis for examining intellectual autonomy. Intellectual autonomy has been identified as an important aim of critical thinking because learners need to retain responsibility for forming, evaluating, and maintaining their own judgments (McPhee and Cox, 2025). Research on generative AI use among Chinese university students has also linked cognitive offloading patterns to self-reported critical thinking and cognitive autonomy, highlighting the importance of metacognitive regulation (Si et al., 2026). Accordingly, the AI-COS does not measure intellectual autonomy directly, but its function-specific dimensions may help researchers examine when AI use involves relatively limited external support vs. greater delegation of evaluative and judgment-related cognitive responsibilities. Future research can compare these dimensions with lower-/higher-order cognitive-demand classifications and dependent/autonomous offloading classifications to determine how cognitive function, cognitive-demand level, and cognitive agency jointly characterize human-AI interaction.

## Conclusions

In sum, the AI Cognitive Offloading Scale for College Students developed in the present study demonstrated satisfactory structural validity, reliability, convergent validity, criterion-related validity, and measurement invariance across gender. The scale comprises three dimensions—memory offloading, assistive offloading, and thinking offloading—and these dimensions can be represented as first-order components of a higher-order AI cognitive offloading construct. At the same time, because formal HTMT or latent-factor correlation statistics were not included in the original analysis, strict statistical discriminant validity among the three highly related dimensions should be examined further in future validation studies.

The findings indicate that college students' AI use encompasses multiple forms of cognitive delegation, including information retrieval and storage, task-related assistance, and judgment or decision support. These forms should not be interpreted as a developmental sequence. Instead, the differences in the magnitude of their associations with AI dependence and metacognitive monitoring suggest that the psychological meaning of AI cognitive offloading may vary according to the function being delegated and the learner's regulation of that delegation. Although all three dimensions were positively associated with AI dependence and negatively associated with metacognitive monitoring, their correlation magnitudes were not identical, and their associations with need for cognition also differed. This pattern highlights the importance of examining the dimensions separately rather than assuming that all forms of offloading are psychologically equivalent.

### Limitations

Although the present study provides relatively comprehensive evidence for the development and validation of the scale, several limitations should be acknowledged.

First, although the sample included a relatively large number of college students, it was still drawn primarily from undergraduate students in a specific contextual setting. Future studies should therefore further examine the external validity of the scale across different regions, types of institutions, and graduate student populations.

Second, the present study employed a cross-sectional design, which does not allow for the examination of dynamic changes in AI cognitive offloading during the learning process, nor does it permit causal inference regarding its antecedents or consequences.

Third, although the current scale distinguishes among memory offloading, assistive offloading, and thinking offloading, the rapid evolution of generative AI may continue to reshape how students use such systems. Future research should therefore revise and refine the items in light of new model capabilities, task contexts, and educational norms.

Fourth, the initial item-development stage was based on literature review, theoretical analysis, and expert content evaluation rather than formal qualitative interviews. Therefore, the present study does not provide interview-derived qualitative evidence for item generation. Future scale-development studies could incorporate systematic open-ended interviews or focus groups, together with detailed coding procedures, to further examine students' lived experiences of AI cognitive offloading and whether additional dimensions or context-specific behaviors should be represented. Combining qualitative exploration with quantitative validation has been illustrated in other domains, such as the development of a multidimensional career-success scale for Chinese employees (Zhou et al., 2013), and offers a methodological example for future scale refinement.

Fifth, future research may also combine interviews, learning logs, or behavioral data to examine whether different forms of AI cognitive offloading correspond to different actual learning outcomes, thereby gradually extending this construct from a self-report measure toward a multi-method assessment framework. Future assessments could also distinguish metacognitive knowledge, monitoring, and control and incorporate task-based measures alongside self-report, drawing on measurement approaches reviewed in educational settings (Rivers et al., 2020).

Sixth, although three dimensions were retained, their coverage of AI cognitive offloading should be examined further across tasks, populations, and contexts. Unexplained item variance may reflect specific variance and measurement error as well as other influences. Existing research suggests that AI cognitive offloading may also vary according to the cognitive-demand level of the delegated task and the manner in which users engage with AI. For example, Jiang and Chen (2026) distinguished lower-order and higher-order AI cognitive offloading and showed that AI evaluation capability may condition associations with these forms, whereas Zhu et al. (2026) distinguished dependent from autonomous offloading according to whether users retain cognitive agency. Future research should therefore examine whether cognitive-demand level, delegation style, AI evaluation capability, task characteristics, and learner characteristics account for additional variance or function as antecedents and boundary conditions of AI cognitive offloading. Longitudinal, experimental, behavioral, and cross-context validation studies may help determine whether these dimensions represent additional components of the construct or complementary dimensions that operate alongside the function-based structure identified in the present study.

Existing reviews also suggest that this field remains in a phase of rapid expansion but uneven evidence development, underscoring the need for structured measurement tools such as the present scale to address the current gap at the level of cognitive processes (Schei et al., 2024; McDonald et al., 2025). A review of early public claims about ChatGPT highlights the need to distinguish expectations about educational transformation from empirical evidence (Jensen et al., 2025). A review of empirical studies further identified heterogeneous approaches and limited shared theoretical grounding in research on generative AI chatbots in higher education (McGrath et al., 2025).

## Data Availability

The raw data supporting the findings of this study are available from the corresponding author upon reasonable request.
